# Supplementary material for: DMRforPairs: identifying Differentially Methylated Regions between unique samples using array based methylation profiles
Source: BMC Bioinformatics. 2014 May 15;15:141. doi: 10.1186/1471-2105-15-141 (PMC4046028; doi:10.1186/1471-2105-15-141)
Supplement: Additional file 2 — DMRforPairs output for the comparison of A431-MCF7 and NA17018-NA17105. Please start from the HTML files in each folder. Available via the BMC Bioinformatics website. [file 1471-2105-15-141-S2.zip › 1394847754114233_MOESM2_ESM/A431_MCF7/figures/20154.pdf]

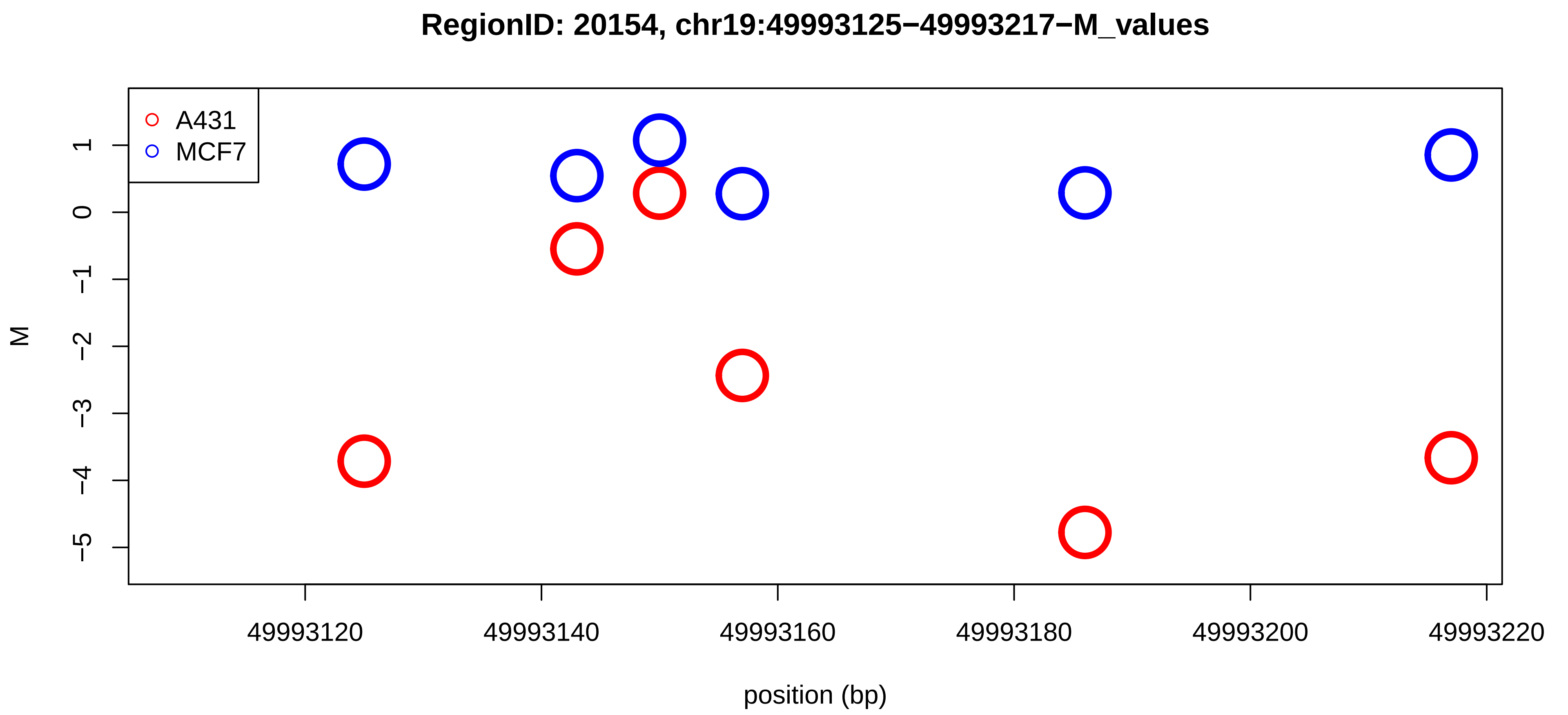
RegionID: 20154, chr19:49993125−49993217−M_values
A431
MCF7
M
1
0
−1
−2
−3
−4
−5
49993120
49993140
49993160
49993180
49993200
49993220
position (bp)

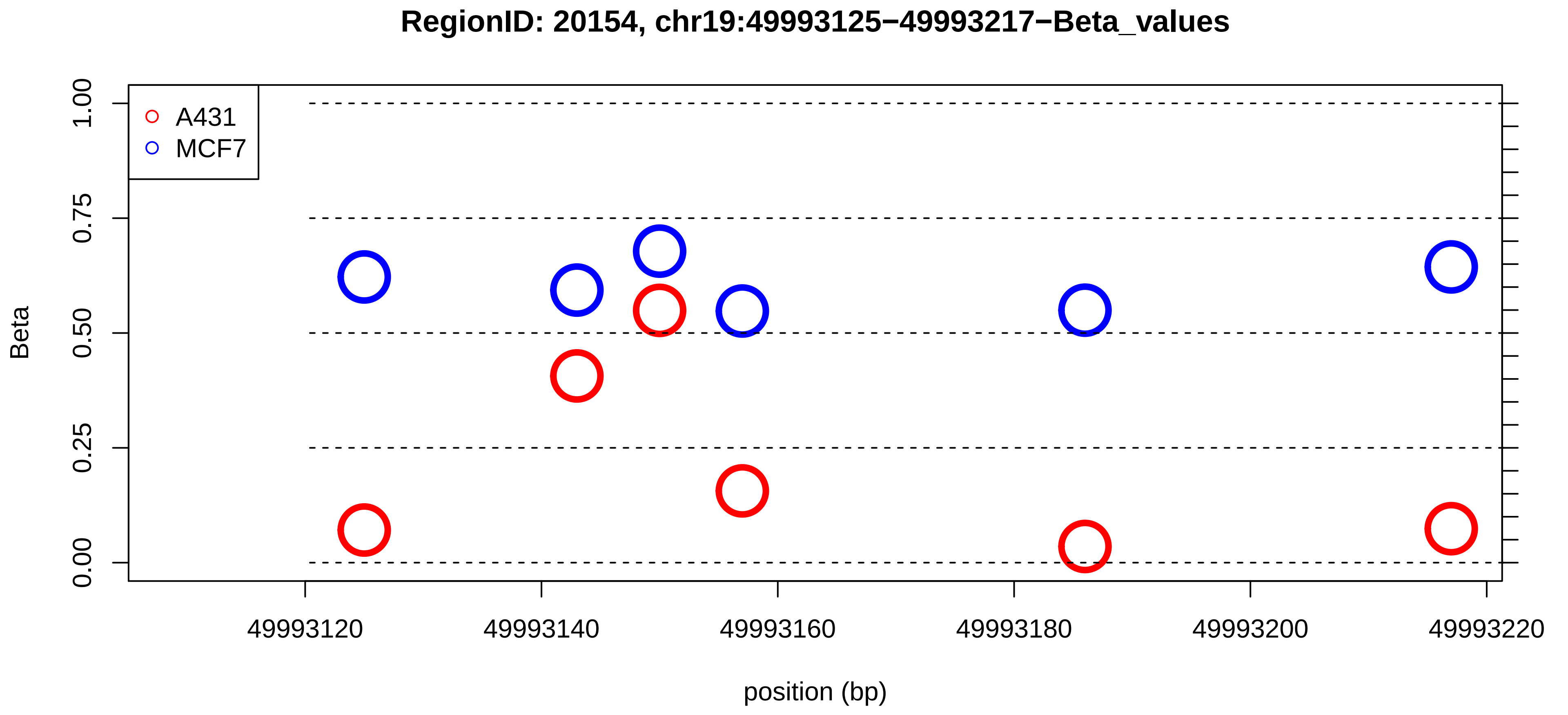
RegionID: 20154, chr19:49993125−49993217−Beta_values
A431
MCF7
Beta
1.00
0.75
0.50
0.25
0.00
49993120
49993140
49993160
49993180
49993200
49993220
position (bp)

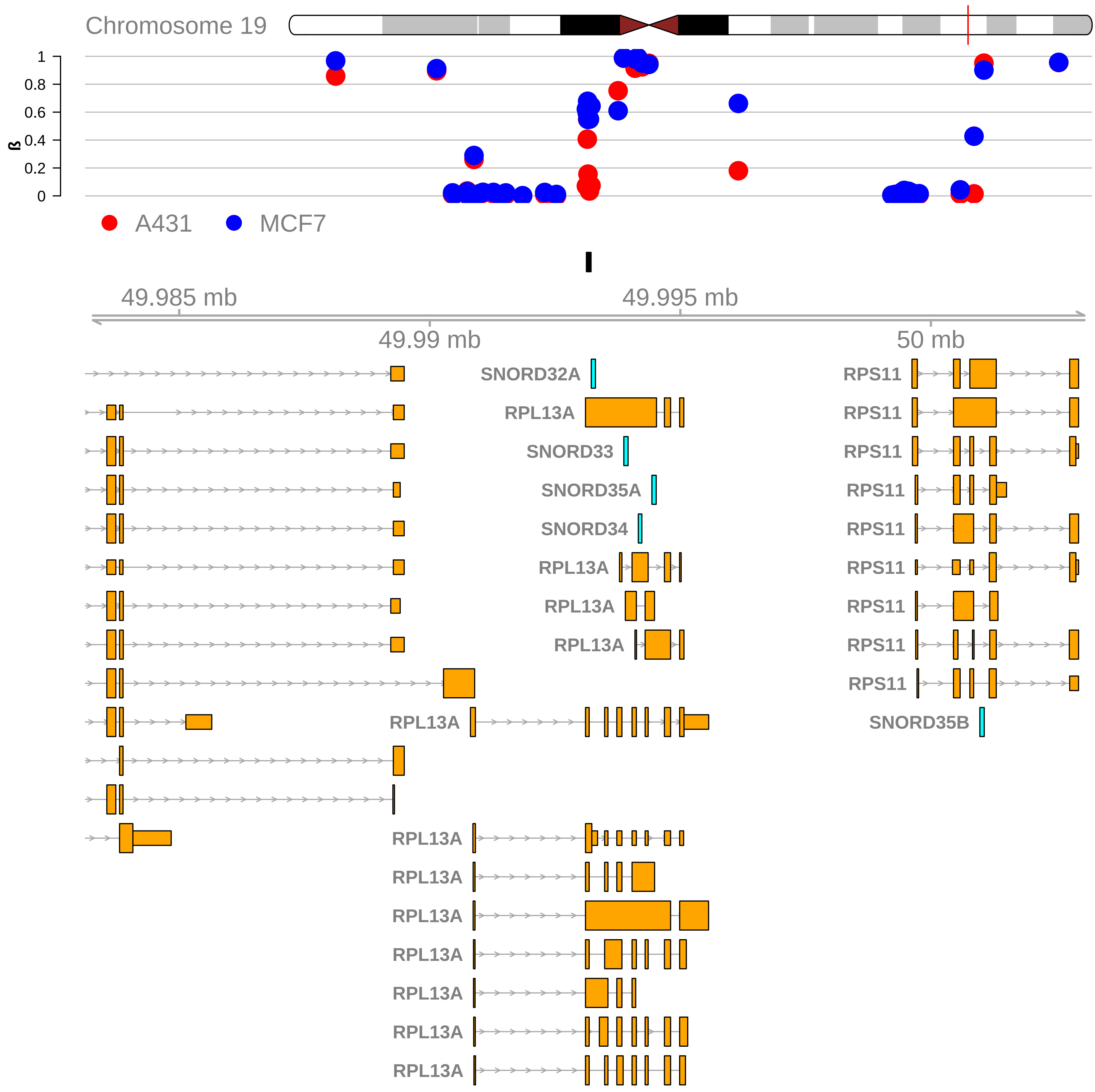
Chromosome 19
ß
1
0.8
0.6
0.4
0.2
0
A431
MCF7
49.985 mb
49.995 mb
49.99 mb
50 mb
SNORD32A
RPL13A
SNORD33
SNORD35A
SNORD34
RPL13A
RPL13A
RPL13A
RPL13A
RPL13A
RPL13A
RPL13A
RPL13A
RPL13A
RPL13A
RPS11
RPS11
RPS11
RPS11
RPS11
RPS11
RPS11
RPS11
RPS11
SNORD35B
